# Improving ART initiation among men who use HIV self‐testing in Malawi: a qualitative study

**DOI:** 10.1002/jia2.25950

**Published:** 2022-06-14

**Authors:** Julie A. Hubbard, Misheck Mphande, Khumbo Phiri, Kelvin Balakasi, Risa M. Hoffman, Joseph Daniels, Augustine Choko, Thomas J. Coates, Kathryn Dovel

**Affiliations:** ^1^ Division of Infectious Diseases David Geffen School of Medicine University of California Los Angeles Los Angeles California USA; ^2^ Partners in Hope Medical Center Lilongwe Malawi; ^3^ Edson College of Nursing and Health Innovation Arizona State University Phoenix Arizona USA; ^4^ Malawi‐Liverpool‐Wellcome Trust Clinical Research Programme TB/HIV Blantyre Malawi; ^5^ University of California Global Health Institute San Francisco California USA

**Keywords:** antiretroviral therapy, highly active, men, qualitative research, self‐testing, sub‐Saharan Africa

## Abstract

**Introduction:**

HIV self‐testing (HIVST) increases HIV testing uptake among men; however, the linkage to antiretroviral therapy (ART) among HIVST users is low. Innovative strategies for ART initiation are needed, yet little is known about the unique barriers to care experienced by male HIVST users, and what ART‐related interventions men desire.

**Methods:**

We conducted semi‐structured in‐depth interviews with cisgender men (≥15 years) in Malawi who tested HIV positive using HIVST between 2018 and 2020, as well as interviews with their female partners (≥15 years) who distributed the HIVST kits. Medical records from seven facilities were used to identify respondents. We included men who received HIVST from a health facility (primary distribution) and from sexual partners (secondary distribution). Interview guides focused on unique barriers to ART initiation following HIVST and desired interventions to improve linkage and initiation. Interviews were audio recorded, translated and transcribed to English, and analysed using constant comparison methods in Atlas.ti v.8.4. Themes were compared by HIVST distribution strategy. Data were collected between 2019 and 2020.

**Results:**

Twenty‐seven respondents were interviewed: eight male/female dyads (16 respondents), eight men without a female partner and three women who represented men who did not participate in the study. Among the 19 men represented (16 men interviewed in person, three represented by secondary report from female partners), seven received HIVST through primary distribution, 12 through secondary distribution. Six men never initiated ART (all secondary HIVST distribution). Barriers to ART initiation centred on the absence of healthcare workers at the time of diagnosis and included lack of external motivation for linkage to care (men had to motivate themselves) and lack of counselling before and after testing (leaving ART‐related fears and misconceptions unaddressed)––the latter was especially true for secondary HIVST distribution. Desired interventions were similar across distribution strategies and included ongoing peer mentorship for normalizing treatment adherence, counselling messages tailored to men, outside‐facility services for convenience and privacy, and facility navigation to help men understand how to navigate ART clinics.

**Conclusions:**

Male HIVST users face unique challenges to ART initiation, especially those receiving HIVST through secondary distribution. Male‐tailored interventions are desired by men and may help overcome barriers to care.

## 1 INTRODUCTION

Traditional HIV testing and treatment efforts continue to miss cisgender men in sub‐Saharan Africa (SSA) [1, 2, 3]. HIV self‐testing (HIVST), whereby an individual tests privately in locations and times convenient to them, has increased testing uptake among men [4, 5, 6, 7] by allowing them to bypass traditional health‐seeking barriers, including time, patient‐level costs and fear of unwanted disclosure in healthcare settings [8]. However, men using HIVST are less likely to link to care and initiate antiretroviral therapy (ART) than those using traditional testing strategies [6, 9, 10, 11]. Current HIVST distribution modalities for men include primary distribution by healthcare workers (HCWs) in clinic and community settings [12, 13, 14, 15, 16]; and secondary distribution by sexual partners [7, 17, 18, 19, 20]. Trials from Malawi and South Africa found that 68% of men using HIVST through primary distribution strategies initiated ART within 3 months [13, 18]; while only 20–28% of those who used HIVST through secondary distribution in trials from Kenya and Malawi initiated ART within the same time period [19, 21].

The convenience, confidentiality and autonomy of HIVST is appealing to men [22, 23, 24], but these benefits may also create unique barriers to initiation. While immediate, same‐day ART initiation has been adopted for routine testing throughout SSA [25], there is currently no provision of a standardized pathway for linkage to care and treatment for those who test HIV positive using HIVST, such as reminders, peer support, or HCW follow‐up. Using HIVST without the presence or proximity of an HCW may exacerbate existing barriers to men's linkage to care, such as fear of unwanted disclosure and HIV stigma, and competing income‐generating demands that make it challenging to attend health facilities [26, 27, 28].

Understanding the unique challenges and potential interventions to support ART initiation among male HIVST users is a critical next step in order for HIVST to be a viable strategy to improve men's engagement in HIV services. We qualitatively examined the barriers to linkage to care and ART initiation for men using HIVST and their preferences for interventions to improve HIV treatment outcomes following self‐testing.

## 2 METHODS

### 2.1 Study setting and participant recruitment

**Figure 1:**
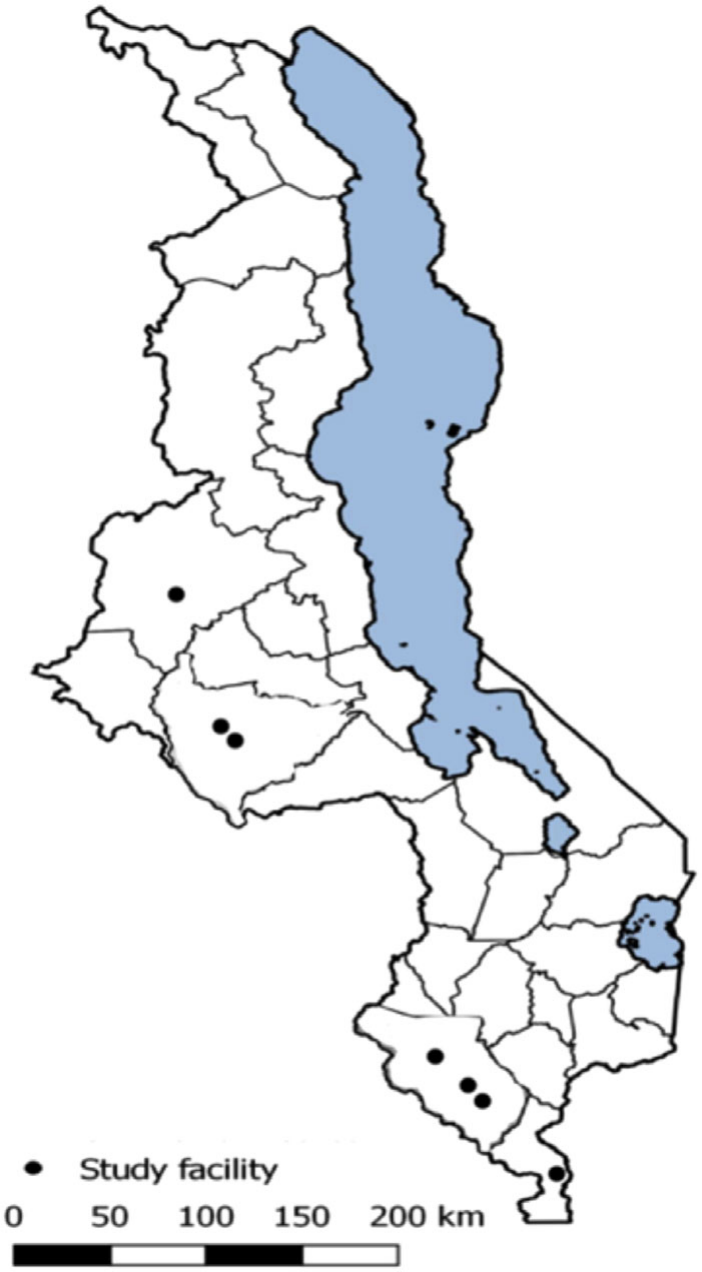
Map of study sites in Malawi (*n* = 7).

This study was conducted in seven high‐burden health facilities in central and southern Malawi where primary and secondary HIVST distribution programs were active (Figure 1). Men who tested HIV positive with HIVST between March 2018 and November 2020 were identified using medical chart reviews and recruited for in‐depth interviews (IDIs) via phone or household visits. Eligibility criteria included: (1) ≥15 years of age; (2) tested HIV positive ≤12 months ago; and (3) personal contact information listed in medical records (i.e. they could be contacted by study staff). Study staff attempted to contact all potential respondents twice within seven days before documenting them as “not found.” Female partners of eligible men were also recruited for separate IDIs to understand their perceptions of their partners’ experience with HIVST and ART initiation. Female partners whose male partners could not be reached or were not able to participate in the study were still eligible for participation.

### 2.2 Data collection

The semi‐structured IDI guide covered the following domains: (1) respondent experiences after testing HIV positive with HIVST (for females, observations of her partners’ experiences); (2) perceived barriers to ART initiation and early retention for male HIVST users; and (3) desired interventions to address barriers, including preferences on the implementation method, time, location and healthcare cadre (for females, perceived ideal interventions for their male partner). Guides were piloted with four respondents (two males and two females) and refined based on feedback. Interviews were conducted by two trained research assistants (one male and one female) in the local language (Chichewa) in quiet, private locations near participating health facilities or the surrounding community. Individuals were paired by gender (i.e. male interviewer with male participant). Interviews were audio‐recorded and lasted 50–90 minutes.

### 2.3 Data analysis

Audio recordings were translated and transcribed to English for analysis, with 20% of transcripts reviewed by an external party to ensure accuracy. An initial codebook was developed based on existing literature on barriers to men's ART initiation [5, 27, 28, 29, 30] and formative work on potential intervention preferences. The codebook was piloted on six transcripts and inductive codes were added. Transcripts were then coded with multiple investigators independently reviewing coding across all transcripts to ensure consistency. Coding and analysis were conducted in Atlas.ti v8.4 [31] using constant comparison methods [32]. Analysis focused on overarching differences or similarities in themes by HIVST distribution strategy (primary and secondary distribution) and men's ART engagement (initiated or not).

### 2.4 Ethical review

Respondents provided oral informed consent and received nine USD to compensate for their time. The study was approved by the National Health Sciences Review Committee in Malawi and the Institutional Review Board at the University of California Los Angeles, USA (Protocol #1664).

## 3 RESULTS

**Figure 2:**
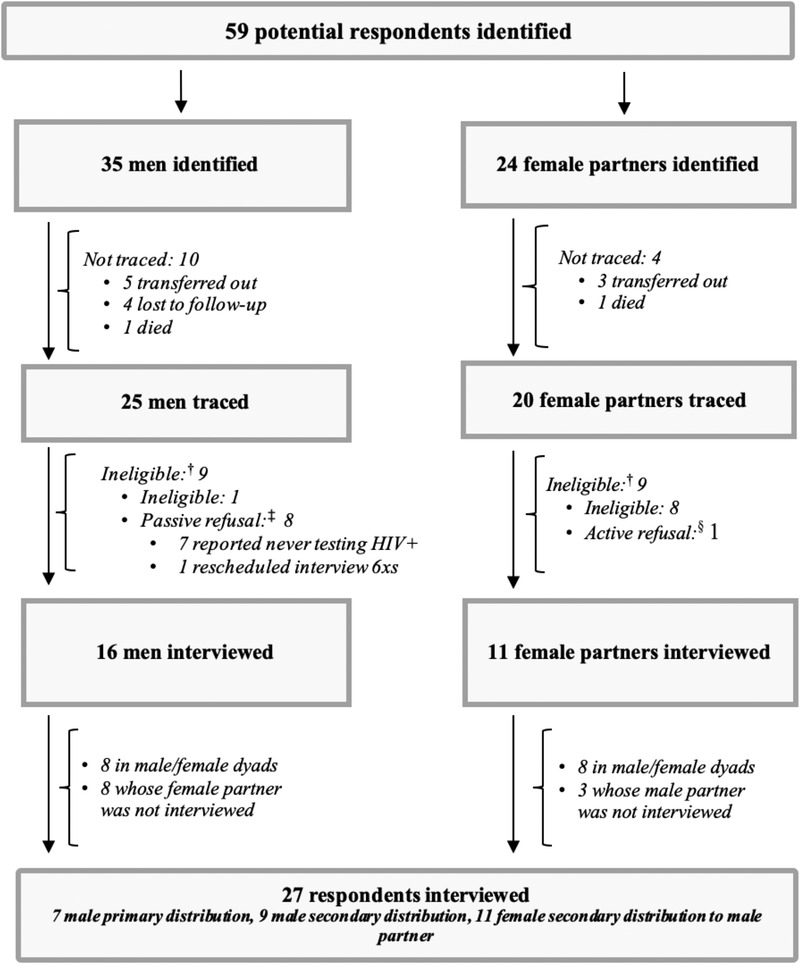
In‐depth interview respondent tracing and recruitment. ^†^ Reasons for ineligibility included age (<15 years) or testing HIV positive >12 months prior to screening. ^‡^ Passive refusal defined as indirect refusal through providing reasons for ineligibility that contradicted medical records or repeatedly requesting interview rebookings. ^§^ Active refusal defined as a direct refusal to participate in study activities.

Data were collected from December 2019 to March 2020 but were temporarily suspended due to the COVID‐19 pandemic. Data collection was resumed from October to November 2020 using the same methodology. Fifty‐nine potential respondents were identified using medical records, of which 45 were successfully traced (Figure 2). Twenty‐seven respondents were interviewed: eight men whose female partners were also interviewed (16 respondents total), eight men who did not have partners or whose partner was unreachable and three women whose partners did not complete an interview.

Men's mean age was 40 years, the majority (*n* = 12/19) were married and half reported having no form of formal employment in the past month (Table 1). Mean age of female partners was 35 years. Among the 19 men represented (including reports from the three females whose male partners did not complete an interview), seven received HIVST from primary distribution at a health facility, 12 from secondary distribution from their female partners. Seven men reported never testing for HIV prior to using HIVST. Among the 19 men represented, 13 men had initiated ART after using HIVST, six had not initiated at the time of interviews.

**Table 1:** Characteristics of interview participants

Variable	Male	Female
	*n* = 16	*n* = 11
**Individual demographics** ^a^		
Age, mean (SD)	40.3 (9.3)	34.9 (5.8)
Attended higher than primary education	18.8 (3)	72.7 (8)
Married	75.0 (12)	100 (11)
Male employment type		
None	37.5 (6)	–
Formally employed^b^	31.2 (5)	–
Self‐employed^c^	25.0 (4)	–
Healthcare worker	6.3 (1)	–
Total number of children, mean (SD)	4.9 (3.1)	3.3 (3.2)
Self‐rated health (very good)	50.0 (8)	36.4 (4)
**HIV and ART initiation variables (*n* = 19)** ^d^		
HIVST distribution type		
Primary distribution	36.8 (7)	–
Secondary distribution	63.2 (12)	–
Previous use of HIV services		
Never tested	43.8 (7)	–
Previously tested HIV+ prior to HIVST use	15.8 (3)	–
Previously on ART prior to HIVST use	0	–
HIV outcomes after HIVST		
Initiated ART within 2 weeks	68.4 (13)	–
Disclosed status to sexual partner	84.2 (16)	–

^a^ All values % (*n*) unless noted otherwise.

^b^ Include security guard, welding, mechanics and carpentry.

^c^ Include farming, fishing and second‐hand clothes.

^d^ Male data include secondary report from women whose male partners did not participate in the study (*n* = 3).

### 3.1 Unique barriers to ART initiation for male HIVST users

While HIVST was readily acknowledged to facilitate testing by promoting privacy and convenience, men described two unique barriers to ART initiation following a reactive HIVST kit: lack of external motivation to support linkage to care (meaning being linked to care by a healthcare worker); and lack of counselling after testing to help them address inaccurate beliefs about HIV and accept their status.

#### 3.1.1 Lack of external motivation to support linkage to care

Both primary and secondary HIVST users described that ART initiation after a reactive HIVST kit was challenging because HCWs were not immediately present to support and guide them into care. Following universal treatment guidelines during routine blood‐based testing [25], HCWs traditionally offer same‐day initiation immediately following a positive test result. In contrast, HIVST users had to seek out ART services themselves––the standard cascade of HCW support, from testing, to counselling for status acceptance, to linkage to care, was broken. Men spoke of the strong personal motivation required to actively pursue additional HIV services on their own:

*You cannot be encouraged by a doctor (when testing alone). You need to motivate yourself. You cannot depend on being pushed, but you have to start for yourself*. (Male initiate, primary HIVST distribution, 41 years)

*When you test with a doctor, whether you like it or not, you start taking medication. I don't think you would run away from it. This is different for one who tests alone*. (Male initiate, primary HIVST distribution, 21 years)

Importantly, primary distribution HIVST users recognized that the personal motivation required for their initiation was less than those who tested outside the facility based on the proximity of HCWs at a health facility.

Female partners frequently described their own attempts to motivate men to link to care after their positive test result; however, many felt ill‐equipped. Female partners of non‐initiates reported they had a limited ability to encourage ART initiation with their partners:

*I encouraged him to go to the hospital to get counseling and start treatment. But he didn't care. He hasn't bothered going to the hospital to get help no matter how hard I tried to talk to him*. (Female partner of non‐initiate, secondary HIVST distribution, 35 years)

*One of my sons offered to escort him on his bicycle and he agreed but … he changed his mind and said ‘I will go the next day’. He never reaches the facility. He gives excuses like, ‘I am not properly dressed so I will go tomorrow.’* (Female partner of non‐initiate, secondary HIVST distribution, 43 years)

#### 3.1.2 Lack of counselling at the time of testing

After a reactive HIVST, most men described a sense of disbelief and shame, coupled with a fear of lifelong medication and unwanted disclosure. Poor ART knowledge was especially apparent for secondary distribution users, many of whom cited inaccurate information about ART side effects as their reason for not initiating. During interviews, some non‐initiates asked study staff, “*What if it (ART) poisons me?*” and “*What if my body becomes saturated (with ART)?”*

Men who participated in primary HIVST distribution received general counselling prior to HIVST distribution but reported seeking out additional counselling on their own after testing positive to address their concerns. Among those who sought out counselling, most said they may not have initiated ART if they had not been provided accurate information and encouragement by an HCW post‐test:

*At home, you debate as to whether you should go to the hospital or stay home. You scare yourself saying, ‘I am already dead. I will not go.’ At the hospital, they have ways to talk to you and calm your fears so that you can start to live a good life. That is the advantage, unlike testing in the village where you would be alone*. (Male initiate, primary HIVST distribution, 45 years)

Women whose partner used secondary distribution HIVST also described that while self‐testing allowed their partner to easily test for HIV, without adequate counselling, the same fears and misconceptions that stopped men from testing at a facility also stop them from linking to care once they knew their HIV status:

*He said he was happy I gave him the kit to test himself because he was afraid of coming to the hospital to get tested. Even now that he has tested positive he still refuses to (initiate)*. (Female partner of non‐initiate, secondary HIVST distribution, 20 years)

### 3.2 Logistical barriers to care mentioned, but overcome

Nearly half of all male respondents reported the inconvenience of attending a healthcare facility after a reactive HIVST kit was a barrier to ART initiation. However, all men who described their health as “poor” prior to testing initiated ART within 1 week after a reactive HIVST. These men attributed their ability to overcome logistical challenges (like travel time and transport costs) to a desire to feel healthy again. This was consistent across both primary and secondary HIVST:

*I was worried (after testing positive) because the distance to the hospital from home is very far in terms of transport … but I was always in pain. That is why I made the decision to come*. (Male initiate, primary HIVST distribution, 35 years)

In contrast, all non‐initiates (all secondary HIVST users) reported feeling healthy at the time of testing. Interestingly, among those who cited facility attendance as a barrier, all but one reported making numerous non‐HIV‐related facility visits for themselves or family members since having a reactive HIVST. None of these men disclosed their HIV status to an HCW during their non‐HIV facility visits, nor were they offered HIV services while receiving other services:

*I may have visited the clinic 8, if not 9 times (since testing positive). I've thought (to start ART) when I was there because whenever I was there I saw sick people and it reminded me that I should start treatment…But the main reason for not starting is I am still healthy*. (Male non‐initiate, secondary HIVST distribution, 34 years)

### 3.3 Desired linkage strategies

Respondents readily identified four interventions that they believed would facilitate ART initiation and early retention among male HIVST users (Table 2).

**Table 2:** Intervention preferences to facilitate ART initiation among male HIVST users as expressed by respondents (*n* = 27)

Intervention	Intervention method	Location	HCW cadre	HCW sex
Peer mentorship by a male mentor living with HIV	In‐person 1‐on‐1, phone or combination	Private location of the respondents choosing	High‐level cadre, community HCW or HIV+ volunteer	Male
Male targeted education and counselling	In‐person 1‐on‐1	Private location of the respondents choosing	High‐level cadre	No preference
Outside facility‐based ART services	1‐on‐1 or group distribution (if private)	“Neutral” locations in the community	High‐level cadre	No preference
Facility navigation	In‐person 1‐on‐1	ART clinic	Community HCW or HIV+ volunteer	Male

#### 3.3.1 Peer support by a male mentor living with HIV

Nearly, all men believed peer mentorship, where men could develop an ongoing relationship with a man who was successfully engaged in HIV care, would encourage ART initiation after using HIVST. Mentors could help men accept their status, address misconceptions about ART, develop strategies for daily adherence and prepare them to visit the ART clinic––bridging the gap left by using HIVST without the presence of an HCW by providing convenient and friendly peer interactions tailored to men's needs.

*With a male mentor (living with HIV), I would have been encouraged (to start ART). I could learn from him since he knows a lot about HIV. To me, that is important to have a man to talk to and learn from. A man is easy to relate to. We can even meet to play Bawo (board game) and discuss my issues*. (Male initiate, primary HIVST distribution, 35 years)

Having support from other males (not females) was an important distinction as many men said they were more comfortable discussing sensitive matters with other men. Some men who initiated ART described already receiving informal support from male friends and family members and emphasized that these relationships were instrumental in their decision to initiate after testing.

*My brother‐in‐law encouraged me by saying, ‘being found HIV positive is not the end of one's life’, and suggesting that I start ART and continue to take the medication. I think of this message all the time, even today*. (Male initiate, primary HIVST distribution, 45 years)

#### 3.3.2 Male‐targeted education and counselling

Both primary and secondary HIVST users desired more counselling at the time of testing but specified that counselling should be tailored to the specific needs and concerns of men. Routine pre‐testing counselling was not enough. Men suggested focusing counselling messages on how ART can practically and *positively* impact men's lives, including men's role as financial providers, men's desire to maintain a strong physical body and men's desire to ensure a strong future for their children. Such topics were described by male initiates as highly motivational in their own decision to initiate:

*What went through my mind (after HIVST) was the children and relatives that I support. I thought if I let fear distract me from starting (ARVs), then there would be challenges for them in the future. I thought if I have more years to live, I can still help them grow and succeed. I was told once that ARVs allow you to do your work just as always. Nowadays you see men going about their farm work and harvesting crops just fine; some are able to have children after diagnosis and the children grow up just fine (without HIV). I asked myself ‘why should I say I am dead when the other people I see are still fine?’ I then told myself to not worry, to accept my status, and take my medication every day*. (Male initiate, primary HIVST, 41 years)

#### 3.3.3 Outside facility‐based services

Most male and female respondents believed providing ART services outside a health facility (i.e. in the community or at home) could improve initiation by increasing convenience, minimizing the risk of unwanted disclosure and––for home‐based HIVST users––“ease” men into HIV treatment services. A handful of participants described already having informal arrangements with HCW's to receive treatment at a private location outside the facility. These men believed that without this informal arrangement, they would not take ART:

*When they [male HCWs] come closer to men, they will get help right away… most [men] want secrecy rather than meeting people at the hospital*. (Male non‐initiate, secondary HIVST distribution, 65 years)

*I have a (male) doctor I call whenever I need my medication. It doesn't take any time. No waiting in crowded lines at the facility*. (Male initiate, primary HIVST distribution, 45 years)

#### 3.3.4 Facility navigation

Finally, many respondents believed that men would benefit from facility navigation during their first ART visit since they may not be familiar with ART clinic protocols. Facility navigation would include explaining ART clinic procedures and teaching them how to navigate the healthcare system. Female respondents were particularly vocal about the need for facility navigation stating that their male partners avoided the ART clinic, in part, because they did not know what to do when there:

*There appears to be no specific spot to be sure of the services one is after. You can be in a waiting line and when you get to the front you are redirected somewhere else without an understanding of what is happening. I would like to know exactly where to go to get the HIV services I want*. (Male initiate, primary HIVST distribution, 35 years)

## 4 DISCUSSION

In this qualitative study, we assessed the unique barriers to ART initiation experienced by men who tested HIV positive with HIVST kits in Malawi, and their desired interventions to support treatment initiation. Barriers specific to HIVST centred around the absence of HCWs during the testing process, which meant that: (1) men had limited external motivators to initiate care (i.e. an HCW was not available to encourage or escort them to treatment); and (2) men received little to no counselling, resulting in persisting fears and myths regarding ART regimens that discouraged initiation. The latter was particularly common among secondary HIVST users. Desired linkage strategies included peer support by a male mentor living with HIV, male‐targeted counselling, outside facility ART services and facility navigation.

HIVST was widely accepted by men because it was private and convenient; however, men described that using HIVST “broke the line of connection” with facility protocols and HCWs in ways that limited external support for ART initiation. Nearly, all men in our study requested some form of additional support (i.e. counselling, education and facility navigation) after receiving a reactive HIVST kit. Emerging literature also finds that men want to be engaged as clients [33]. These findings contradict common preconceptions that men are difficult clients who do not want to be reached, or who want a “hands‐off approach” to HIV treatment [34]. Such stereotypes harm men by justifying little to no tailored services for men as clients, resulting in insufficient support for men to engage in care [35, 36, 37, 38]. On the other hand, there is also extensive literature showing that men (and women) do not want to be “forced” or coerced into using HIV services––HCWs must engage men as equals and agents of their own healthcare [39, 40]. Together, these findings highlight the importance of client‐centred, client‐empowering models of care for men that provides support for treatment engagement without coercing them into care [41].

While logistics were mentioned as a primary barrier to ART initiation for male HIVST users, men were able to overcome such barriers when the benefit of initiation clearly outweighed the cost (i.e. for those who were in poor health, returning to good health is worth the time and travel required to start treatment). Importantly, all non‐initiates in our study felt healthy at the time of testing. Other studies also find that feeling healthy may reduce one's internal motivation to start treatment [27]. External encouragement and counselling may be particularly critical for healthy men since ART initiation may not have immediate, noticeable benefits to their perceived health.

We found that most male HIVST users desired male‐specific counselling or tailored, ongoing peer mentorship from another male who was successfully engaged in HIV services. Another study on linkage strategies following HIVST in Uganda also found that “peer‐leaders” were the most acceptable individuals to provide follow‐up and linkage support as they were seen to be trustworthy [42]. Similarly, a meta‐analysis found that community testing strategies with facilitated linkage, or follow‐up by counsellors/peers, achieved the highest rates of ART initiation as compared to those without facilitated linkage [9]. Our findings add to this literature and suggest that the use of *male* peer mentors could further improve acceptability among male HIVST users. For secondary HIVST distribution, this finding is of great significance as female sexual partners are traditionally the sole support available to men at the time of testing within index testing models. While there are numerous peer counselling and mentorship programs tailored to adolescents [43] and mothers living with HIV [44], there are very few led by men for men [33]. Using counselling materials tailored to men's specific needs, such as fear of unwanted disclosure, perceived stigma and how to manage ART visits with being a wage earner [35], could further maximize impact.

Outside‐facility ART dispensing was appealing to male HIVST users––especially men who tested at home. Differentiated models of care (DMOC) have largely targeted stable clients (i.e. adherent on ART > 6 months). However, there is shifting interest in developing DMOCs for patients in their first 6 months of care [45, 46], and preliminary evidence shows that the provision of home‐based dispensing is a beneficial strategy to support initiation among HIVST users [47]. Dispensing ART in the community for the first or multiple refills may help “ease” men into care, providing a pathway to facility‐based care.

In addition, non‐HIV clinics (such as outpatient departments) can take simple steps to support linkage among those who access non‐HIV services [48]. Integrating routine HIV screening and offering non‐judgmental, male‐specific counselling regarding early ART initiation (and re‐initiation) in outpatient settings may help HIV programs reach men who know their status but have not initiated ART. The majority of men who did not initiate ART reported attending outpatient departments at health facilities more than once since testing HIV positive, either for their own acute health (non‐HIV related) or the health of others. Other studies in Malawi show high facility attendance among the general male population (82% within the past 12 months) [49].

This study has several limitations. First, seven of the 25 men contacted for the study said they had never tested HIV positive. There is little reason to believe that medical charts were incorrect, suggesting that these men may have been living with HIV but did not want to interact with the study staff. Barriers and desired interventions for ART initiation likely differ for this important sub‐population. Second, not all participants were a part of a dyad that was willing or able to participate. We believe the secondary reports from female partners whose partners could not be reached or refused to complete a survey represent an important hard‐to‐reach group of men, and thus their reports were included in the analysis. Further research specific to understand secondary distribution and linkage may consider conducting dyadic analysis. Finally, findings may not be generalizable outside Malawi. Additional research is needed from other settings, especially for secondary HIVST distribution users who may face the greatest barriers to ART initiation.

## 5 CONCLUSIONS

HIVST appeals to men due to the flexibility and privacy it offers. However, interventions to ensure a pathway to initiation following testing are lagging, leaving men with little guidance for the next steps towards care. Our study revealed a range of service delivery preferences voiced by male HIVST users that may optimize the HIVST strategy.

## COMPETING INTERESTS

None declared.

## AUTHORS’ CONTRIBUTIONS

KD and TJC conceptualized the study. KD is responsible for funding acquisition. JAH, MM, KP, KB and KD developed the study protocol and in‐depth interview guides. JAH and MM implemented the study. JAH, MM and KD developed the analysis plan and codebook. JAH coded and analysed the data with support from KD, JD, RMH and AC. JAH wrote the first draft, and MM, KP, KB, RMH, JD, AC, TJC and KD edited following drafts. All authors have read and approved the final manuscript.

## FUNDING

This work is supported by the Bill and Melinda Gates Foundation (INV‐001423). KD was supported by NIMH R01‐MH122308, Fogarty International K01‐TW011484‐01 and UCLA GSTTP.

## Data Availability

The authors confirm that the data supporting the findings of this study are available within the article. Full qualitative transcripts are available on request from the corresponding author.
